# Ultrasound triggered topical delivery of *Bmp7* mRNA for white fat browning induction via engineered smart exosomes

**DOI:** 10.1186/s12951-021-01145-3

**Published:** 2021-12-04

**Authors:** Yitong Guo, Zhuo Wan, Ping Zhao, Mengying Wei, Yunnan Liu, Te Bu, Wenqi Sun, Zhelong Li, Lijun Yuan

**Affiliations:** 1 Department of Ultrasound Diagnosis, Tangdu Hospital, Fourth Military Medical University, Xi’an, 710038 China; 2 Department of Hematology, Tangdu Hospital, Fourth Military Medical University, Xi’an, 710038 People’s Republic of China; 3 State Key Laboratory of Cancer Biology, Department of Biochemistry and Molecular Biology, Fourth Military Medical University, Xi’an, 710032 China

**Keywords:** Targeted drug delivery, Exosomes, Stealth technique, Ultrasound, Bmp7, White fat browning

## Abstract

**Background:**

Efficient and topical delivery of drugs is essential for maximized efficacy and minimized toxicity. In this study, we aimed to design an exosome-based drug delivery platform endowed with the ability of escaping from phagocytosis at non-target organs and controllably releasing drugs at targeted location.

**Results:**

The swtichable stealth coat CP05-TK-mPEG was synthesized and anchored onto exosomes through the interaction between peptide CP05 and exosomal surface marker CD63. Chlorin e6 (Ce6) was loaded into exosomes by direct incubation. Controllable removal of PEG could be achieved by breaking thioketal (TK) through reactive oxygen species (ROS), which was produced by Ce6 under ultrasound irradiation. The whole platform was called SmartExo. The stealth effects were analyzed in RAW264.7 cells and C57BL/6 mice via tracing the exosomes. To confirm the efficacy of the engineered smart exosomes, Bone morphogenetic protein 7 (*Bmp7*) mRNA was encapsulated into exosomes by transfection of overexpressing plasmid, followed by stealth coating, with the exosomes designated as SmartExo@Bmp7. Therapeutic advantages of SmartExo@Bmp7 were proved by targeted delivering *Bmp7* mRNA to omental adipose tissue (OAT) of obese C57BL/6 mice for browning induction. SmartExo platform was successfully constructed without changing the basic characteristics of exosomes. The engineered exosomes effectively escaped from the phagocytosis by RAW264.7 and non-target organs. In addition, the SmartExo could be uptaken locally on-demand by ultrasound mediated removal of the stealth coat. Compared with control exosomes, SmartExo@Bmp7 effectively delivered *Bmp7* mRNA into OAT upon ultrasound irradiation, and induced OAT browning, as evidenced by the histology of OAT and increased expression of uncoupling protein 1 (*Ucp1*).

**Conclusions:**

The proposed SmartExo-based delivery platform, which minimizes side effects and maximizing drug efficacy, offers a novel safe and efficient approach for targeted drug delivery. As a proof, the SmartExo@Bmp7 induced local white adipose tissue browning, and it would be a promising strategy for anti-obesity therapy.

**Graphical Abstract:**

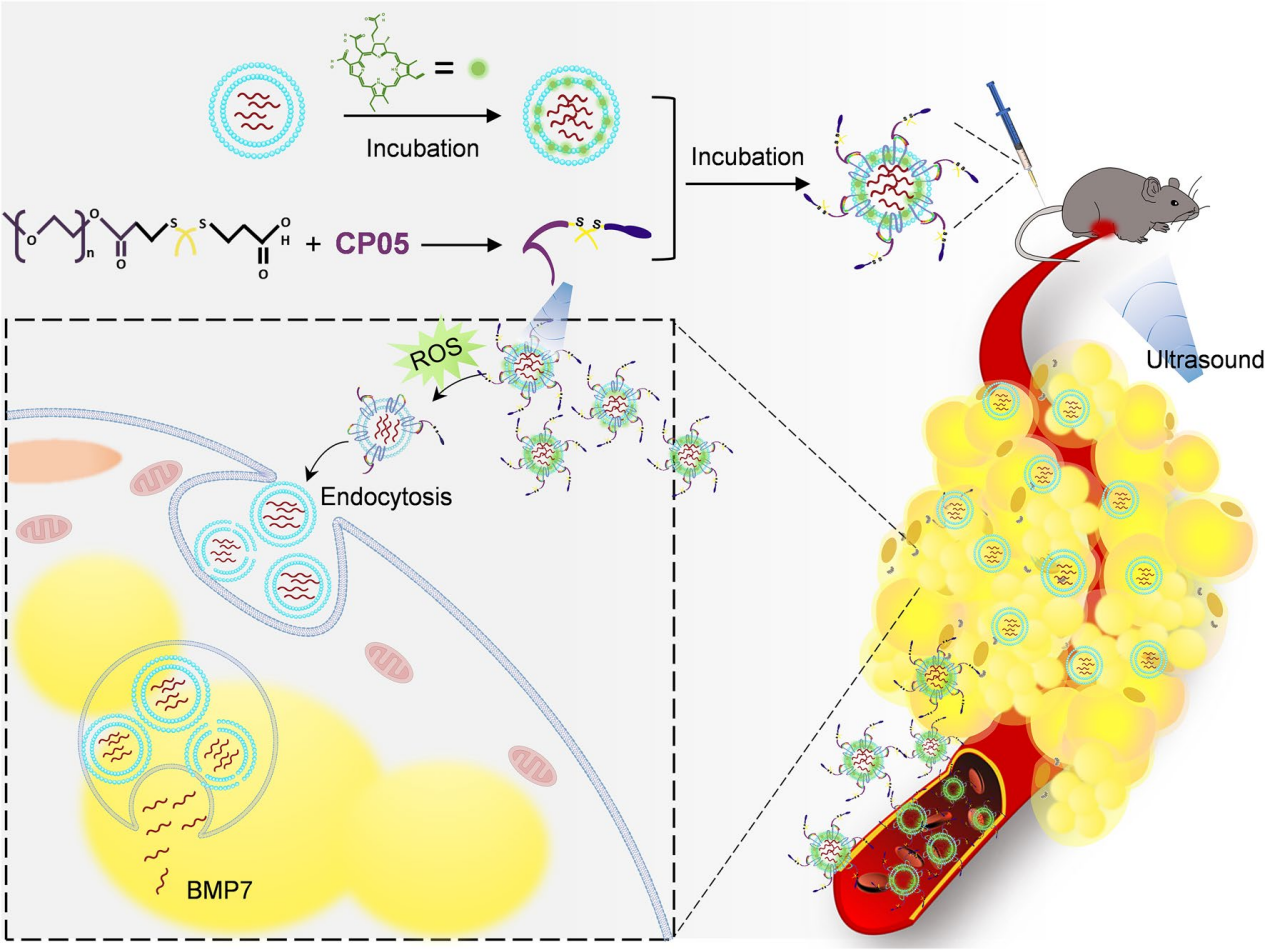

**Supplementary Information:**

The online version contains supplementary material available at 10.1186/s12951-021-01145-3.

## Background

Exosomes are a type of nanovesicles, 30–150 nm in diameter, that have the same topology as the parental cells and are enriched in proteins, lipids, nucleic acids and glycoconjugates [1]. In recent years, exosomes have emerged as a promising platform in drug delivering system. Despite the significant advantages in low immunogenicity, biocompatibility, ability of targeting different tissues with their surface ligands, etc., most of the exosomes have been found distributed into liver, spleen and other tissues with mononuclear phagocyte system (MPS), limiting their therapeutic efficacies [2–6].

Intensive efforts have focused on fusing targeting peptides and other moieties to exosomal surface to improve the delivering efficiency [7–9]. However, those strategies are challenged as some targeting ligands are refractory to proper expression and prone to degradation [10]. Alternatively, the targeting delivery efficiency is significantly rely on overexpression of targeting peptides, which can be difficult to control and may lead to massive side effects [7–9]. In addition, unlike the synthetic materials, decorated exosomes cannot thoroughly escape from MPS since its good biocompatibility [7–9]. Therefore, to realize the controllable and targeting delivery of therapeutic molecules using exosomes as drug carriers, a smart enough exosome capable of escaping of MPS is urgently required.

It is well known that the hydrophilic polymer polyethylene glycol (PEG) corona, i.e., PEGylation, is an efficient strategy to protect drug carriers from aggregation, opsonization, and phagocytosis, consequently prolonging their circulation time and decreasing collateral damage to healthy tissues [11, 12]. It has been reported that reactive oxygen species (ROS) could readily cleave thioketal (TK) bonds hence expose the drug carrier in core by removing the TK linked corona [13]. Herein, we propose a smart exosomes-based delivery system constructed with a novel conjugate designated as CP05-TK-mPEG and a sonosensitizer chlorin e6 (Ce6). In this system, CP05 is the peptide, CRHSQMTVTSRL, with potent capacity bound to tetraspinin CD63 [14], allowing PEG anchored onto exosomes. Moreover, Ce6 was loaded in exosomes through direct incubation. The smart exosomes could escape from phagocytosis and locally deliver drugs on-demand by ultrasound.

As a proof-of-concept study, we confirmed that smart exosome-based system could deliver *Bmp7* locally and induce the local omental adipose tissue (OAT) browning efficiently, serving as a promising strategy for anti-obesity therapy.

## Results

### Identification and characterization of exosomes

**Fig. 1:**
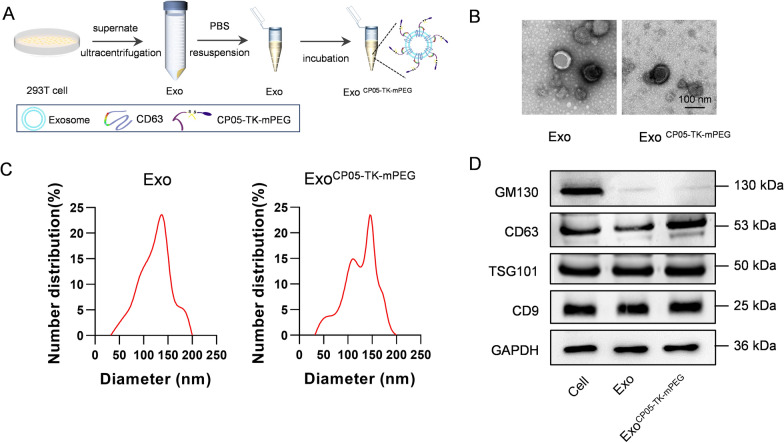
Construction and characterization of Exo^CP05−TK−mPEG^. **A** Illustration of construction of Exo^CP05−TK−mPEG^. **B** TEM images of Exo and Exo^CP05−TK−mPEG^. **C** Size distribution of Exo and Exo^CP05−TK−mPEG^. **D** Western blot analysis of exosome markers in parental cells, Exo and Exo^CP05−TK−mPEG^

CP05-TK-mPEG was anchored onto exosomes by direct incubation (Fig. 1A).To characterize CP05-TK-mPEG functionalized exosomes (Exo^CP05−TK−mPEG^), morphology analysis through transmission electron microscope, particle size distribution analysis by dynamic light scattering and exosomal marker expression by western blot were applied. Transmission electron microscopy verified the shape and size of Exo^CP05−TK−mPEG^ were nearly the same as Exo (Fig. 1B). Particle size analysis showed that the size of Exo^CP05−TK−mPEG^ was slightly larger than the parental Exo, with whose size fell into the diameter range of 30–160 nm (Fig. 1C). As expected, CP05-TK-mPEG functionalization didn’t change the expression profile of the inclusive markers (CD9, TSG101 and CD63) and exclusive marker GM130 (Fig. 1D).

### Evidence of Escaping from Phagocytosis of Exo^CP05−TK−mPEG^ in vitro and in vivo

**Fig. 2:**
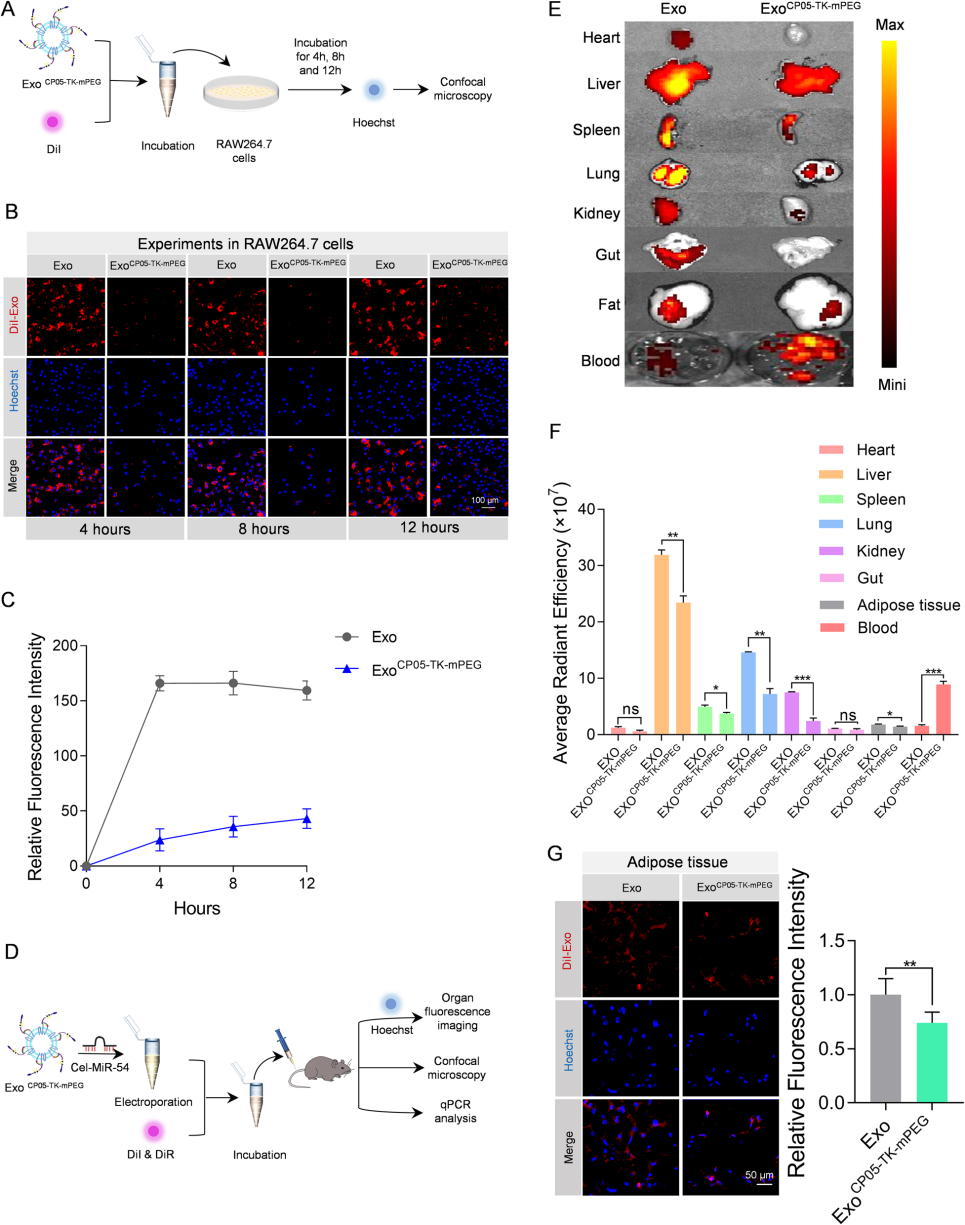
Exo^CP05−TK−mPEG^ escape from phagocytosis both in vitro and in vivo. **A** Schematic illustration of the in vitro experimental procedures. Exo^CP05−TK−mPEG^ was stained with DiI and put into Raw 264.7 cells incubating for 4 h, 8 h, and 12 h for confocal images. **B** Representative confocal images of DiI-labeled exosomes (red) in RAW264.7 cells. The nuclei were counter-stained with Hoechst (blue). DiI-labeled exosomes were added to RAW264.7 cells and incubated for 4 h, 8 h, and 12 h before fluorescence microscopy. **C** Relative fluorescence intensities of the confocal images at 4 h, 8 and 12 h. **D** Schematic illustration of the in vivo experimental procedures. Exo ^CP05−TK−mPEG^ was loaded with cel-miR-54 then stained with DiI or DiR for organ fluorescence imaging, confocal microscopy and qPCR analysis. **E** Representative fluorescence showing the localization of DiR-labeled exosomes in various organs and blood in mice treated with Exo and Exo ^CP05−TK−mPEG^. **F** Average radiant efficiency for above images. **G** Representative confocal images of DiI-labeled exosomes (red) in abdominal adipose tissue of mice. The nuclei of adipose cells were counter-stained with Hoechst (blue). The relative fluorescence intensity was shown at right panel. All data are expressed as mean ± SEM. *p < 0.05, **p < 0.01, ***p < 0.001 between groups. Error bars represent the SEM for n = 3

In order to determine the ability of escaping from phagocytosis, Exo and Exo^CP05−TK−mPEG^ were incubated with cells for different hours to observe the phagocytosis (Fig. 2A). Compared to the control group, there were much less Exo^CP05−TK−mPEG^ uptaken in different incubation period (Fig. 2B, C), suggesting that Exo^CP05−TK−mPEG^ effectively escaped from endocytosing.

To visualize and compare the organ distribution of Exo^CP05−TK−mPEG^, Exo and Exo^CP05−TK−mPEG^ were labeled with fluorescent dye under dark conditions and then injected into mice (Fig. 2D). As expected, Exo^CP05−TK−mPEG^ were less concentrated in major organs and tissue, including heart, liver, spleen, lung, kidney, gut and abdominal adipose tissue, while more concentrated in circulating blood compared to natural Exo (Fig. 2E, F). In addition, confocal fluorescence imaging also evidenced the ability of escaping from phagocytosis of Exo^CP05−TK−mPEG^ in above organs (Additional file 1: Fig. S1). Notably, Exo^CP05−TK−mPEG^ has just mild ability of escaping from taken up by the abdominal adipose tissue (Fig. 2G). For further evidence, Exo and Exo^CP05−TK−mPEG^ were electroporated with cel-miR-54 (Fig. 2D) followed by in vivo injection. qPCR analysis revealed that the expression level of cel-miR-54 is significantly lower in Exo^CP05−TK−mPEG^ than that in Exo in most of the observed organs (Additional file 1: Fig. S2), which was consistent with fluorescence tracking results.

### Construction of SmartExo and ultrasound triggered local removal of PEG corona

**Fig. 3:**
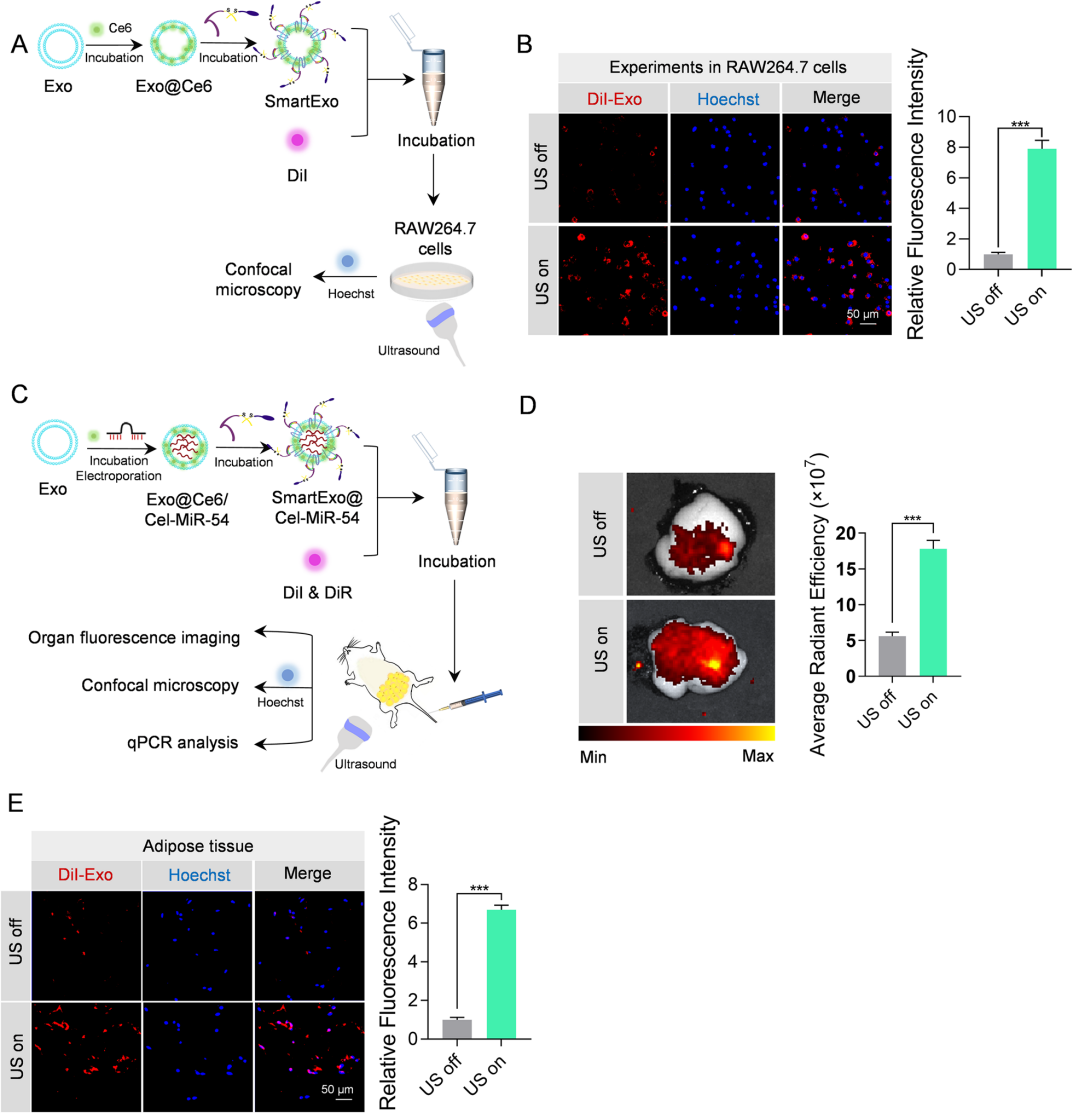
Focused ultrasound removes PEG corona from the SmartExo and induced phagocytosis in the target organ. **A** Schematic illustration of the in vitro experiments. Ce6 and PEG decorated exosomes were stained with DiI an put into RAW264.7 Cells. The phagocytosis of exosomes with and without irradiation of ultrasound was observed and compared by confocal microscopy. **B** Representative confocal images of DiI-labeled exosomes (red) in RAW264.7 cells. The nuclei were counter-stained with Hoechst (blue). DiI-labeled exosomes were added to RAW264.7 cells and ultrasound irradiation was applied or not applied. The relative fluorescence intensity was shown at the right side. **C** Decorated exosomes were loaded with cel-miR-54 to evidence the phagocytosis at target organ with or without ultrasound irradiation, and ultrasound irradiation was applied once a hour in the first 6 h after injection. **D** Representative confocal images of DiI-labeled exosomes (red) in abdominal adipose tissue of mice with or without ultrasound irradiation. The average radiant efficiency is at right side. **E** Representative fluorescence showing the phagocytosis of DiR-labeled exosomes by abdominal adipose tissue with or without ultrasound irradiation. Relative fluorescence intensity of confocal images was shown at the right side

The above data suggest that Exo^CP05−TK−mPEG^ prolongs the circulation time in blood while removal of the PEG is prerequisite for boost local delivery. To this end, Exo were incubated with Ce6 followed by incubation with CP05-TK-mPEG, with the exosome system designated as SmartExo. SmartExo was then DiI-labeled and incubated with RAW264.7 cells for 30 min. Ultrasound was applied to produce ROS by the sonodymamic effects of Ce6, which would theoretically cleave the TK bond and free exosomes from PEG corona (Fig. 3A). The confocal images showed that under ultrasound on (US on) condition, the effect of phagocytosis was much stronger than that in ultrasound off (US off) condition (Fig. 3B). Moreover, in vivo experiments showed that ultrasound irradiation significantly improved the delivery of SmartExo in adipose tissue (Fig. 3C–E). Cel-miR-54 tracing by qPCR analysis further confirmed the findings (Additional file 1: Fig. S3).

### Construction of SmartExo@Bmp7 for targeted delivery of *Bmp7* mRNA

**Fig. 4:**
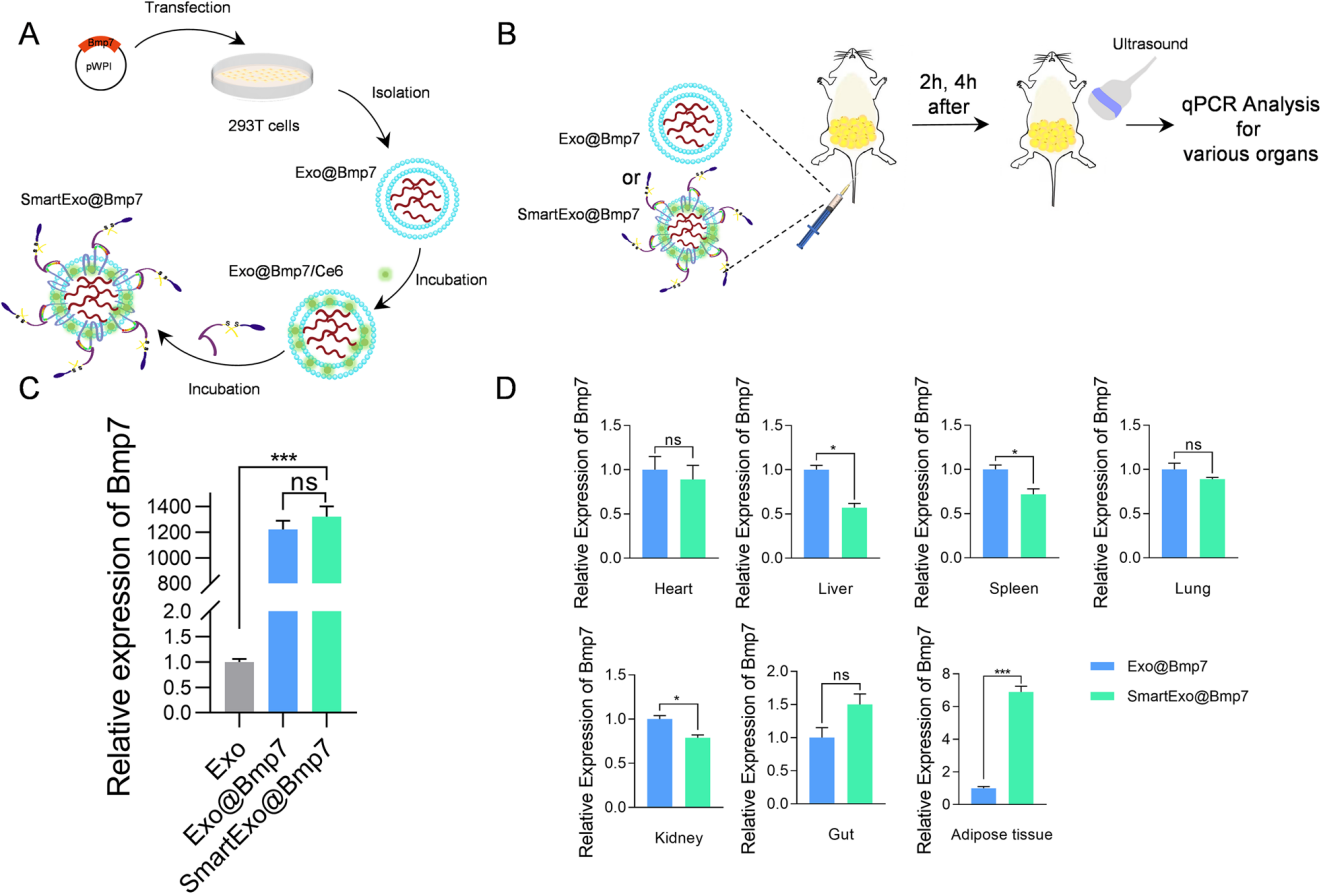
Construction of ultrasound controllable smart exosome-based system for *Bmp7* targeting delivery. **A** Schematic illustration of exosome-based *Bmp7* delivery system SmartExo@Bmp7 construction. **B** Schematic illustration of comparison of delivery efficiency of Exo@Bmp7 and SmartExo@Bmp7. **C** qPCR analysis of relative expression level of Bmp7 in exosomes isolated from control and BMP7 transfected 293T cells (Exo@Bmp7) and SmartExo@Bmp7. **D** Relative expression of *Bmp7* mRNA in various organs. Mice were treated with Exo@Bmp7 and SmartExo@Bmp7 and additionally treated with ultrasound irradiation in the abdominal region. Data are expressed as mean ± SEM, n = 3, *p< 0.05, ***p < 0.001

Currently, the whole world is struggling with a pandemic of obesity and seeking for efficient therapeutic strategies for its treatment is a constant need [15]. Researchers have found that the amount of brown adipose tissue (BAT) is positively in correlation with the energy expenditure and is significantly lower than in slim adults [16]. Among the strategies aimed at induction of BAT, the bone morphology protein 7 (*Bmp7*) is an important inducer of brown adipocyte differentiation [17, 18]. Both in vitro and in vivo studies have evidenced that *Bmp7* could induce fully differentiated brown adipocytes with high uncoupling protein 1 (*Ucp-1*) expression [19, 20]. In the following experiments, we aimed to deliver *Bmp7* mRNA with the above SmartExo system. Breifly, plasmid overexpressing *Bmp7* mRNA was transfected in 293T cells, and after 48 h exosomes loaded with *Bmp7* (Exo@Bmp7) were isolated by ultracentrifugation. Then Exo@Bmp7 was functionalized to SmartExo@Bmp7 using the method constructed above (Fig. 4A). For verification of delivery specificity in abdominal adipose tissue, Exo@Bmp7 and SmartExo@Bmp7 were injected into mice via tail followed by ultrasound induction on abdominal adipose tissue, and qPCR analysis of *Bmp7* mRNA expression of various organs was applied (Fig. 4B). As expected, Bmp7 mRNA was efficiently loaded into exosomes while SmartExo engineering had no obvious effects on the abundance (Fig. 4C). In addition, relative expression of *Bmp7* in adipose tissue was significantly increased, while that in other organs were slightly decreased or not significant (Fig. 4D). Accordingly, there was a significant increase of BMP7 protein expression in adipose tissue of mice treated with SmartExo@Bmp7 (Additional file 1: Fig. S4).

### Smart exosome-based system applied in *Bmp7* targeting delivery for OAT browning

**Fig. 5:**
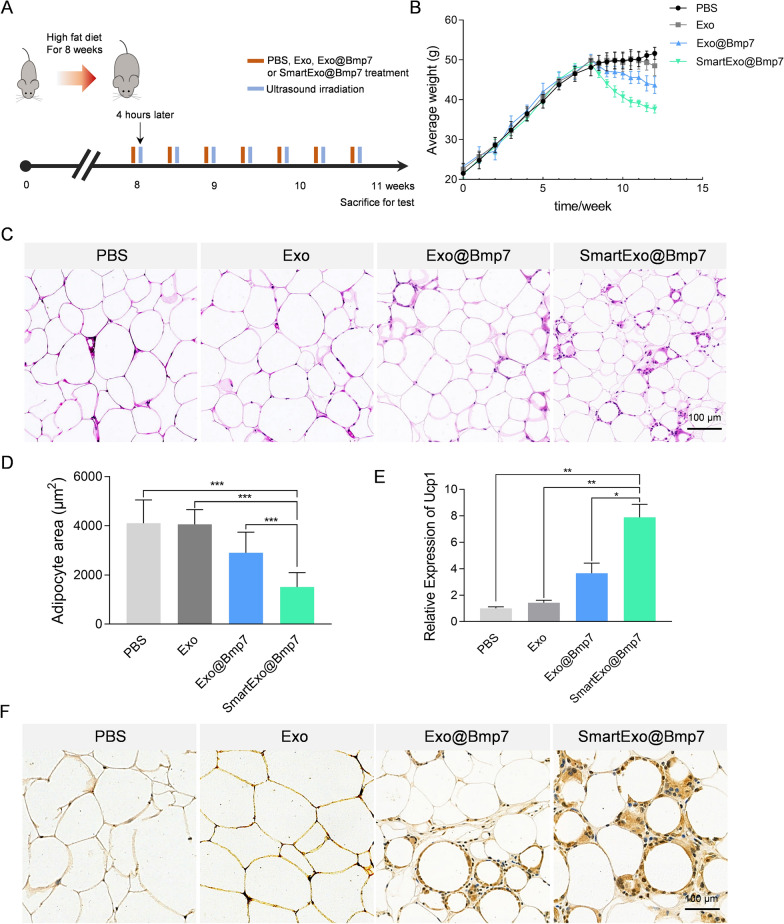
The efficiency of ultrasound induced smart exosome-based system delivering *Bmp7* for OAT browning. **A** Schematic diagram of the experimental procedure. Treatment time points of PBS, natural exosomes (Exo), exosomes loaded with *Bmp7* (Exo@Bmp7) or smart exosome-based system loaded with *Bmp7* (SmartExo@Bmp7) and the application of ultrasound for all groups. **B** Average weight of each group recorded starting from the high fat diet to the end of treatment period. **C** Representative images of HE staining of OAT from C57BL/6 male mice intraveneously treated with PBS, Exo, Exo@Bmp7 or SmartExo@Bmp7. **D** Relative area of lipid droplets for HE staining images. **E** Relative expression level of *Ucp1* in OAT from C57BL/6 male mice intraveneously treated with as PBS, Exo, Exo@Bmp7 and SmartExo@Bmp7. Gapdh served as an internal control and data are expressed as mean ± SEM. **p < 0.01, ***p < 0.001 between groups. Error bars represent the SEM for n = 3. **F** Representative images of *Ucp1* staining of section of OAT depots from C57BL/6 male mice intraveneously treated with PBS, Exo, Exo@Bmp7 and SmartExo@Bmp7

In view of the above data, we aimed to explore whether SmartExo@Bmp7 induce adipose browning in obese mice. Mice were fed in high-fat-diet for induction of obesity. After 8 weeks of feeding, models of obesity were successfully built considering the weight was at least 20% more than the normal-chow-diet ones. The obesity mice were randomly allocated to PBS, Exo, Exo@Bmp7 or SmartExo@Bmp7 treatment group. The exosome treatment was performed one every three day for 3 weeks. Ultrasound irradiation was conducted once every 1 h in the first 6 h after tail vein injection (Fig. 5A). It was evidenced that average body weight of the group treated with SmartExo@Bmp7 significantly dropped, while the Exo@Bmp7 treated group slightly dropped, in comparison with other groups (Fig. 5B). Consistently, the weight of OAT in group treated with SmartExo@Bmp7 was much lower than all other three groups (Additional file 1: Fig. S5). Histology examination of OAT revealed prominent decrease of adipocyte size after SmartExo@Bmp7 treatment (Fig. 5C, D). Accordingly, the relative of expression of *Ucp1* in OAT of SmartExo@Bmp7 treated mice was significantly higher than other groups (Fig. 5E). Immunohistochemical staining of adipose tissue for *Ucp1* further confirmed that SmartExo@Bmp7 induced browning of OAT (Fig. 5F).

## Discussion

We here developed a smart exosome-based drug delivery strategy by coating CP05-TK conjugated PEG to the surface of exosomes. In the meantime, sonosensitizer Ce6 was loaded into exosomes. The resultant exosomes (SmartExo) successfully prolonged the circulation time in blood shown as escaping phagocytosis from major organs. Moreover, since Ce6 was loaded in those exosomes, with the intervention of ultrasound at the location of target organ, the redox reaction between ROS, which was produced by Ce6, and TK freed exosomes from PEG corona so that it can be eaten off properly by the target organ. In vitro and in vivo experiments revealed that the system exerts the ability of escaping from phagocytosis at non-target sites and controllable releasing drugs at right organs. Last but not the least, using SmartExo system, with irradiation of ultrasound at abdominal region, *Bmp7* was successfully delivered on site which was shown as OAT browning. The results showed that SmartExo system was a great prospect of targeting delivering strategy, and its efficiency was proved by targeting delivery of *Bmp7* on OAT for obesity treatment.

Natural exosomes are of poor cell- or tissue- specific targeting features [8]. Thus, the therapeutic application of exosomes as a delivery platform necessitates the design of controlled and targeted exosomes to promote therapeutic efficacy [7, 21, 22]. Previous researches showed that the targeting function of exosomes can be achieved by either parental cell-based engineering or direct exosome engineering, displaying specific proteins or RNAs on the surface or into the lumen of exosomes so that it can be identified by target organs or tissue [4, 22, 23]. Although the targeting peptides or proteins of exosomes could deliver molecules to specific cells, it is not avoidable that phagocytosis happened on non-target organ [24]. Therefore, a prospective exosome-based delivery platform would be capable of both evasion of phagocytosis on non-target sites and controllable release drugs in desirable places.

Researchers have found that PEG coatings on nanoparticles, i.e., PEGylation, shielded the surface against aggregation, opsonization and phagocytosis, and it was a common approach for improve the circulation time of nanoparticles in vivo[11, 12]. Combining the requirement of targeting delivery, we thought a switchable PEGylation of exosomes would hold great promise for shielding them from interaction with cells and improving drug delivery efficiency. Recently, a particular peptide, CP05, was found of capable of direct painting of exosomes irrespective of the origin of exosomes through specifically binding to the second extracellular loop of CD63 [14]. In the light of that, we thought PEGylation of exosomes could be accomplished by construction of a conjugate in which PEG corona wrapped exosomes through CP05 anchored on CD63. In order to get rid of the PEG protection allowing phagocytosis of exosomes at target organs, ROS-sensitive covalent bonds can be introduced as a part of conjugate [25]. In our study, thioketal was used as a link between CP05 and PEG forming CP05-TK-mPEG. Under the assistance of photosensitizers or sonosensitizers, lights or ultrasound generates ROS, which readily cleave thioketal. In fact, thioketal could be replaced with phenylboronic ester, diselenide bonds, or any other ROS-sensitive non-cytotoxic covalent bonds if it is possible [26].

Both light and ultrasound irradiation can trigger the respective sensitizers producing ROS. Although the sonodynamic therapeutic is developed from photodynamic therapeutic, ultrasound is more applicable than laser [27]. Unlike laser, ultrasound trigger sound-sensitizer by sound waves under certain wave length with deeper penetration [27, 28]. According to previous researches, ultrasound is capable of penetrating into deep tissue, about tens of centimeters, allowing treatment with deep nidus or interaction with sonosensitizer in deep site [29]. In addition, ultrasound treatment has good repeatability since it is nonradiative and costs lower than laser application [27]. As a representative photo/sonosensitizer, Ce6 has been extensively used in photo/sonodynamic treatment due to its high singlet oxygen generation efficiency [30]. In our study, Ce6 was chosen as the sonosensitizer loaded in exosomes and triggered by ultrasound producing ROS in order to cleave TK.

As far as we have known, current drugs for weight loss approved by the FDA focus primarily on the reduction of energy intake, and few of them provides adequate long-term clinical efficacy [31]. BAT is essential for thermogenesis and energy balance in mammals, and in vivo experiments’ results revealed its promotion for energy expenditure, reduction of adiposity, and protecting mice from diet-induced obesity [15, 32, 33]. According to previous studies, the mesodermal growth factor *Bmp7* can stimulate BAT activation and promote the energy expenditure reducing weight [19, 34]. However, given the fact that *Bmp7* is also recognized as a cytokine with pleiotropic functions being offered as a treatment for cardiovascular, fibrotic, metabolic and neurodegenerative diseases, systematic administration of *Bmp7* could bring unexpected side effects [35–39]. In our study, *Bmp7* was loaded in SmartExo system and induced at topical OAT assisted by ultrasound. Since the exosomes derived from HEK293T cells were reported as having high biocompatible and low immunogenicity, the *Bmp7* engineered exosomes induced no significant inflammatory response (Data not shown) [40]. This strategy guarantees the browning of local white adipose tissue with little leakage of *Bmp7* to other organs. This newly smart exosome-based delivering strategy of *Bmp7* could provide new directions in the development of therapeutics for obesity.

## Conclusions

In summary, we here have established an ultrasound-assisted and smart exosome-based delivery system, namely SmartExo, which delivers the drugs to the local area. In contrast to most drug targeting delivery mechanism which concentrate on expressing of various peptide or moieties that can vary between tissues or organs, SmartExo provides a controllable delivery platform based on ultrasound, regardless of the type or targeting location, thus offering a robust and broadly applicable targeting strategy. With further optimization, the SmartExo-based targeting delivery strategy described here might be applied in the context wherever prevention of non-target phagocytosis and promotion of drug releasing at target organs are badly needed.

## Methods

### Cell culture

HEK293T cells and RAW 264.7 cells were cultured in complete media containing high glucose Dulbecco’s modified Eagle medium (DMEM) (Hyclone, US) with 10% fetal bovine serum (Exocell, China) and 1% penicillin/streptomycin (Hyclone, US) in a humidified incubator with 5% CO2 at 37 °C.

### Plasmid construction

*Bmp7* cDNA was synthesized in Genscript and subcloned into pWPI vector using PacI and BstB1, with the resultant correct clone designated as pWPI-*Bmp7*. All the clones were confirmed by sequencing and the right clones were stored at − 80 °C for following application.

### Transfection

In order to load *Bmp7* mRNA into exosomes, HEK293T cells were transfected with the corresponding plasmid with Lipofectamine 2000 (Invitrogen) following standard instruction. In brief, culture medium was replaced with antibiotic-free DMEM medium when cell density grew to 60–70% in 100-mm culture dish. Then, 20 µL Lipofectamine 2000 and 10 µg plasmid were incubated separately in two centrifuge tubes containing 500 µL of DMEM medium at room temperature for 5 min. After that, contents of two centrifuge tubes were mixed and incubated at room temperature for 20 min. The above mixture was added to HEK293T cell culture dish mentioned before.

### Exosome isolation and purification

The HEK293T cells were used as the exosome donor cells in this study. Cell culture supernatants were replaced with serum-free medium at least 48 h before isolation process. The supernatants were collected and differentially centrifuged (250*g* for 10 min at 4 °C to eliminate dead cells and 10,000*g* for 15 min at 4 °C to remove residual cellular debris) followed by filtration (0.22 μm filter; Milipore-Sigma, Bilicera, MA). Next, the resulting supernatant was centrifuged at 100,000*g* and 4 ℃ for 2 h to obtain the exosomes. Finally, the extracted exosomes were re-suspended in PBS and stored at − 80 °C before using.

### Transmission electron microscopy

The morphology of isolated exosomes was analyzed by transmission electron microscopy. Briefly, the exosome suspension was added onto the copper mesh, then the exosomes were stained with 2% uranyl acetate for 1 min and imaged by the electron microscope (JEM-2000EX TEM, JEOL Ltd, Tokyo, Japan).

### Particle size analysis

For measure the size of distribution, the isolated exosomes were diluted to 1 µg/mL and the measurement was performed and analyzed using NanoPlus (Otsuka Electronics, Japan). Measurements were repeated at least three times for each sample.

### Determination of concentration of exosomes by BCA method

The concentration of suspended exosomes was determined by total protein concentration. A small aliquot of exosomes was dissolved RIPA Lysis Buffer. Determination of exosomal protein concentration was performed by Pierce BCA Protein Assay Kit (Thermo, USA) under standard instruction [7–9].

### Western blotting

Total proteins from cells or exosomes were extracted at 4 °C for 30 min by using RIPA Lysis Buffer (Beyotime, China). Adipose tissue samples were homogenized using a Teflon/glass homogenizer in lysis buffer with pH 7.4, containing 50 mM Tris-HCL buffer, 250 mM NaCl, 5 mM EDTA, 0.1% Triton X-100, 50 mM NaF, 1 mM orthovanadate, and protease inhibitors. Then, the lysates were centrifuged at 500*g* for 10 min at 4 °C and the supernatant was collected. Western blotting was performed according to the concentration of proteins determined by BCA method. Primary antibodies used in this study included anti-GM130 (1:1000, Abcam, ab30637), anti-CD63 (1:1000, Abcam, ab134045), anti-TSG101 (1:500, Santa, sc-7964), anti-CD9 (1:1000, Abcam, ab236630), anti-GAPDH (Proteintech, 60004-1-1 g) and anti-BMP7 (1:1000, Abcam, ab129156). Secondary antibodies were HRP-conjugated goat anti-mouse IgG (D110087, BBI, China) and HRP-conjugated goat anti-rabbit IgG (1:5000, Cell Signaling Technology, 7074P2).

### Synthesis of CP05-TK-mPEG

About 100 mg of Maleimide-Thioketal-PEG2000 (Mal-TK-mPEG) were dissolved in 5 mL *N*,*N*-dimethylformamide (DMF), then, 1.1 eq of the peptide CRHSQMTVTSRL (CP05) was added to the solution and incubated at room temperature for 12 h. After that, the mixture was loaded into dialysis tubing with molecular cut off at 3500 Da, and the dialyse in purified water for 24 h. Finally, the dialysate was collected and the final product CP05-TK-mPEG was obtained using freeze-drying method.

### Construction of SmartExo

Ce6 (diluted in DMSO, 30 mg/mL ) were encapsulated into exosomes (1 µg/µL ) through electroporation. Then, Ce6 loaded exosomes were isolated and resuspended with CP05-TK-mPEG solutions (1 mg CP05-TK-mPEG powder dissolved in 1 mL PBS) and incubated at 4 °C overnight. The decorated exosomes were isolated and washed twice using PBS to remove residuals, and the products were resuspended in PBS as SmartExo.

### Cel-miR-54 loading into exosomes

The cel-miR-54 (GenePharma, China) sequence is listed in Additional file 1: Table S2. Exosomes were loaded with cel-miR-54 through electroporation. Briefly, 100 µg exosomes were mixed with 0.5 OD cel-miR-54 mimics in 4 mm electroporation cuvette (BioRad, USA) and electroporated on Gene Pulser Xcell^TM^ Total System (BioRad, USA) at 700 V/150 µF. After electroporation, the mixture were placed on ice for at least 30 min to recover the membrane.

### In vitro tracing of exosomes

Exosomes (about 1 µg/µL of protein concentration) were labeled with DiI o by incubation with the dye (1 mM) at the ratio of 500:1 for 30 min in 37 °C dark room, followed by exosome isolation as described above. RAW 264.7 cells were incubated with DiI-labeled exosomes for 4 h, 8 h, and 12 h. For those were incubated with DiI-labeled SmartExo, ultrasound was applied after 40 min of incubation. The ultrasound irradiation intensity was set as 0.1 W/cm^2^ with a duty cycle of 20%, and the irradiation time was 30 s (Additional file 1: Table S1). The cells were then washed with PBS for three times and fixed with 4% paraformaldehyde for 10 min and again washed with PBS twice. Cell nuclei were counter-stained with 1 µg/mL Hoechst 33,342 (Beyotime, China) diluted at 1:1000 for 10 min in 37 °C dark room then washed with sodium acetate solution to remove the nonspecific adhesion. The prepared samples were observed and captured by Confocal Microscope (Eclipse C2, Nikon, Japan).

### Animal experiments

Healthy male C57/BL6J mice aged 8 weeks were purchased from Animal Center of the Fourth Military Medical University. The animal experimental and housing procedures were performed in accordance with the protocols of Animal Experimentation and Ethics Committee of Fourth Military Medical University.

### In vivo fluorescence tracing of exosomes

Exosomes (about 1 µg/µL of protein concentration) were labeled with DiI or DiR by incubation with the dye (1 mM) at the ratio of 500:1 for 30 min in 37 °C dark room. Organ distribution analysis of exosomes was performed as previously described. Briefly, DiR-labeled exosomes were washed with PBS to remove free dyes and tail-vein injected into mice. The dosages of exosomes treatment vary from study to study, and 4 µg/g of exosomes per mouse for each time was chosen in our study, which is similar as the dose in published literatures [21]. For those were injected with SmartExo, ultrasound was applied on the abdominal area once every hour in the first 6 h after injection. For in vivo experiments, the irradiation intensity was set as 2 W/cm^2^, and the duration was 180 s for each irradiation (Additional file 1: Table S1). The distribution of exosomes in organs was detected 8 h after injection using an IVIS Lumina II in vivo imaging system (PerkinElmer, Thermo Fisher, US).

For test of the sliced section, exosomes labeled with fluorescent dye DiI (Beyotime, China) were administered into mice intravenously. Eight hours after the injection or ultrasound, the fresh organs were harvested, then washed with PBS and fixed in 4% paraformaldehyde for 30 min before being embedded in optimal cutting temperature compound. Then, the embedded organs were sliced into 10 μm sections on a freezing microtome (Leica, Germany). Tissue sections were stained with 1 µg/mL Hoechst 33,342 (Beyotime, China) diluted at 1:1000 for 10 min then washed with PBS before sealing with neutral gum under dark conditions. The prepared samples were observed and captured by Confocal Microscope (Eclipse C2, Nikon, Japan) for exosomes tracking.

### Anti-obesity therapy

All mice were fed with high-fat-diet and body weights of mice were monitored weekly. At the 8th week, mice fed a high-fat-diet developed obesity (weighing at least 20% more than mice fed with normal chow diet). All animals were randomly allocated to four groups (four mice for each group) to start treatments for obesity once three days for totally 3 weeks. The first group was treated with physiological saline solution (PBS), the second group was treated with natural exosomes (Exo, the third group was treated with *Bmp7* loaded exosomes (Exo@Bmp7) and the last group was treated with *Bmp7* loaded smart exosome-based drug delivery system (SmartExo@Bmp7). The dosage of exosomes in each group was determined as 4 µg/g per mouse as mentioned above. Ultrasound irradiation was applied on the abdominal area once every hour in the first 6 h after injection for each group in order to remove as much as PEG corona from SmartExo. The parameters were set same as described above (Additional file 1: Table S1). All animals were sacrificed after 3 weeks of treatments, and abdominal fat were obtained to evaluation the browning situation.

### RNA isolation and qRT-PCR

Total RNA of the sample was extracted using TRIzol (Invitrogen, 15596018) followed the standard instructions. For mRNA, 2 µg of RNA was reverse transcribed into cDNA by SMART® MMLV Reverse Transcriptase (Takara). While, for miRNA, 2 µg of RNA was reverse transcribed into cDNA using miRcute Plus miRNA qPCR detection kit (Tiangen). Experiment of qPCR was performed with FastStart Essential DNA Green Master Kit (06924204001, Roche). Relative expression of mRNA and miRNA was respectively normalized to *GAPDH/Gapdh* and *U6* levels and calculated by 2^−ΔΔCt^. The PCR primer sequences are supplied in Additional file 1: Table S2.

### Histology

OAT were excised and fixed in 10% formalin. Then they were paraffin-embedded and sectioned prior to H&E staining or immunohistochemistry for *Ucp1* (Abcam; 1:400) detection. Signal was detected using the Vector ABC Elite kit.

### Statistical analysis

Data and results are expressed as mean ± SEM as indicated. Student’s t-test was used for comparison between two groups (Graphpad Prism 7.0). P values of < 0.05 indicate statistical difference.

## Supplementary Information

**Additional file 1: Figure S1.** Representative confocal images of DiI-labeled exosomes (red) in various organs of mice. **Figure S2.** Expression of cel-miR-54 in various organs receiving exosomes as indicated. **Figure S3.** Expression level of cel-miR-54 in OAT with or without ultrasound irradiation. **Figure S4.** Western blot analysis of Bmp7 in adipose tissue of mice treated with Exo, Exo@Bmp7 and SmartExo@Bmp7. **Figure S5.** Average OAT weights of mice treated with PBS, Exo, Exo@Bmp7 and SmartExo@Bmp7. **Table S1.** Ultrasound irradiation parameters. **Table S2.** Primers used in the study.

## Data Availability

The datasets used and/or analyzed during the current study are available from the corresponding author on reasonable request. Additional information available: additional Figs. S1–S4 and Tables S1, S2.

## Abbreviations

PEG: Polyethylene glycol
TK: Thioketal
Ce6: Chlorin e6
ROS: Reactive oxygen species
Bmp7: Bone morphologenetic protein 7
Ucp1: Uncoupling protein 1
OAT: Omental adipose tissue
BAT: Brown adipose tissue
MPS: Mononuclear phygocyte system
