# Supplementary material for: Ultrasound triggered topical delivery of Bmp7 mRNA for white fat browning induction via engineered smart exosomes
Source: J Nanobiotechnology. 2021 Dec 4;19:402. doi: 10.1186/s12951-021-01145-3 (PMC8645082; doi:10.1186/s12951-021-01145-3)
Supplement: Supplementary file 1 — Additional file 1: Figure S1. Representative confocal images of DiI-labeled exosomes (red) in various organs of mice. Figure S2. Expression of cel-miR-54 in various organs receiving exosomes as indicated. Figure S3. Expression level of cel-miR-54 in OAT with or without ultrasound irradiation. Figure S4. Western blot analysis of Bmp7 in adipose tissue of mice treated with Exo, Exo@Bmp7 and SmartExo@Bmp7. Figure S5. Average OAT weights of mice treated with PBS, Exo, Exo@Bmp7 and SmartExo@Bmp7. Table S1. Ultrasound irradiation parameters. Table S2. Primers used in the study. [file 12951_2021_1145_MOESM1_ESM.docx]

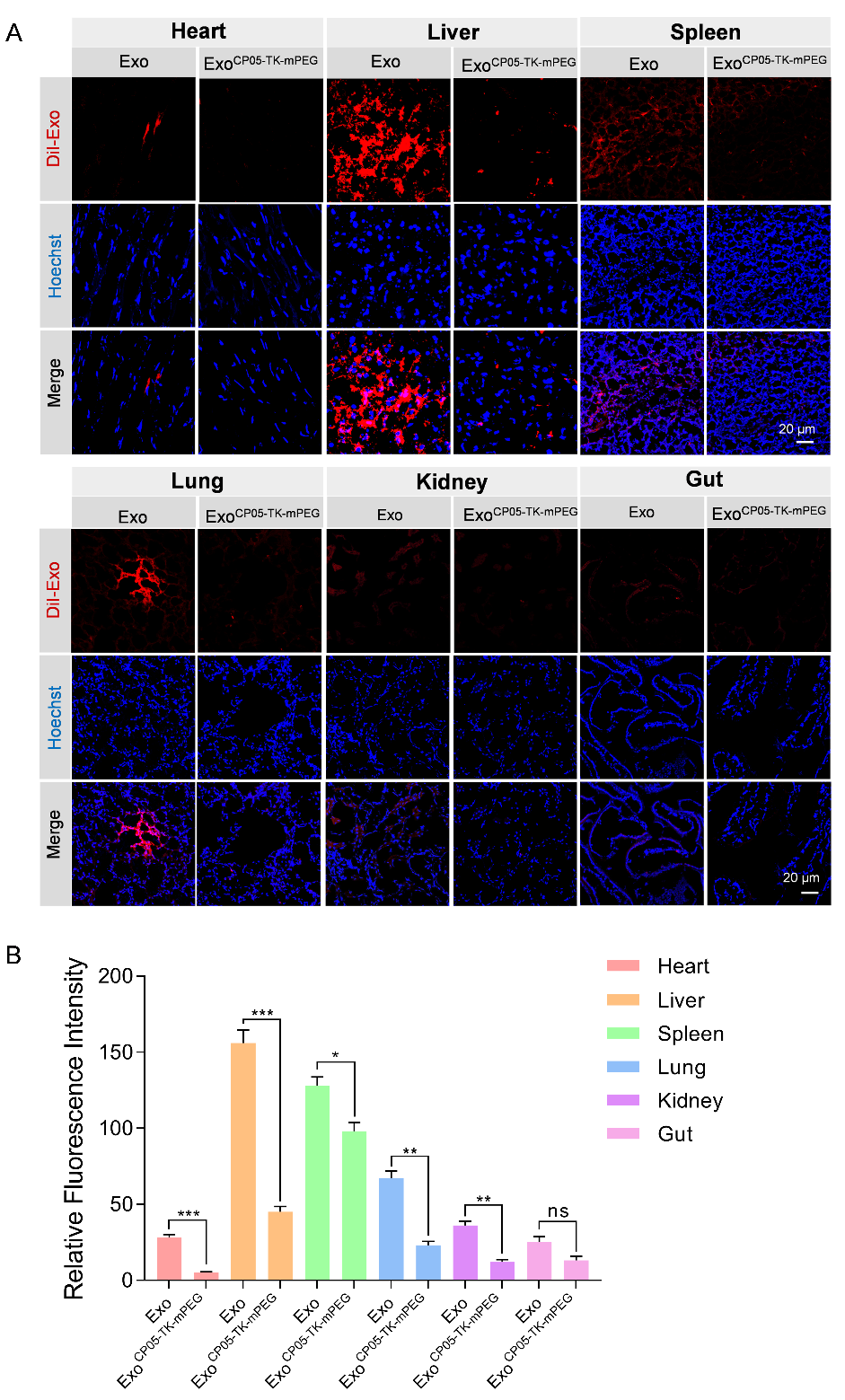
**Supplemental figures**

**Figure S1. Representative confocal images of DiI-labeled exosomes (red) in various organs of mice.** (A)The nuclei of adipose cells were counter-stained with Hoechst (blue). (B) The relative fluorescence intensity of confocal images in A. All data are expressed as mean ± SEM. * p<0.05, ** p<0.01, *** p<0.001 between groups. Error bars represent the SEM for n=3.


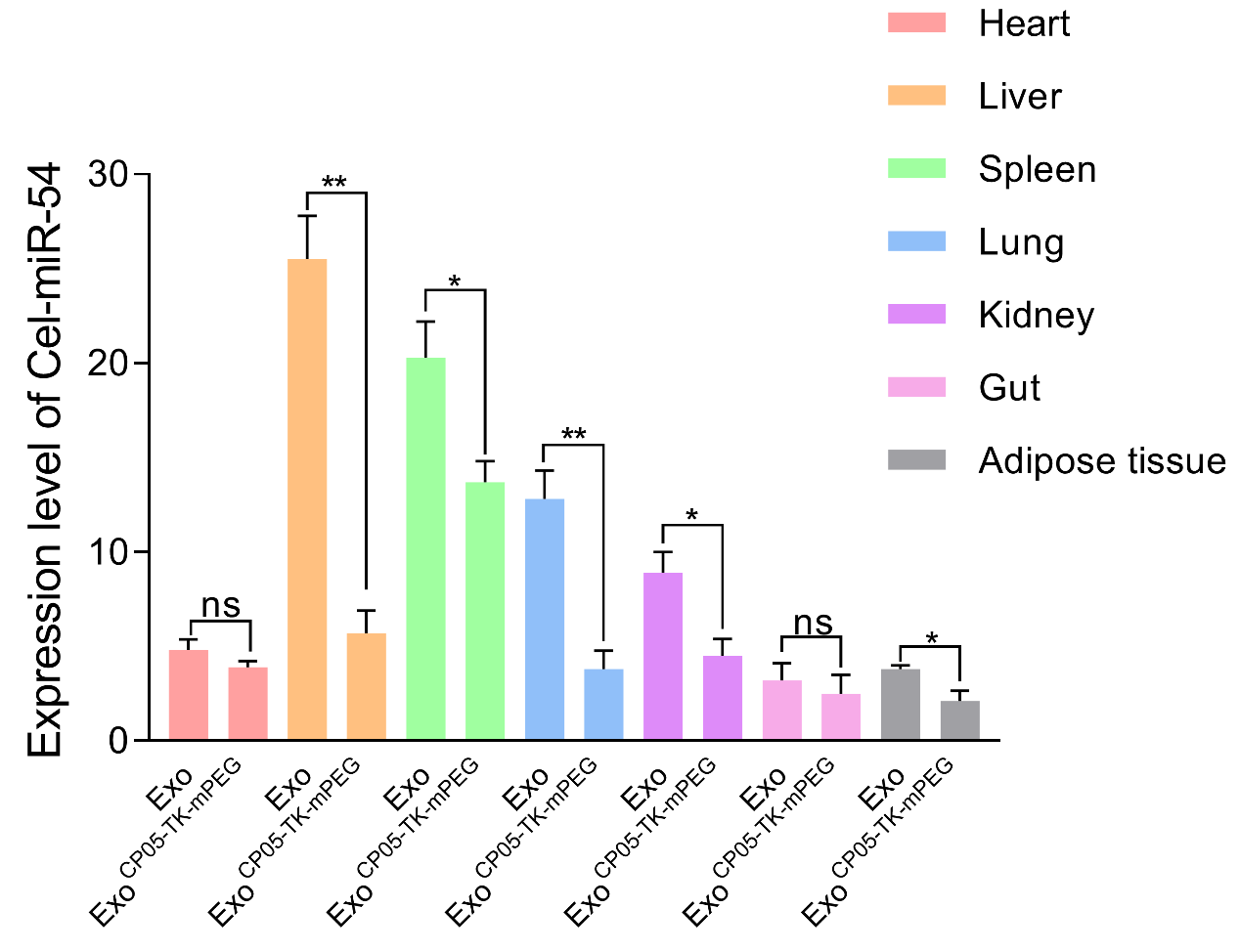


**Figure S2 Expression of cel-miR-54 in various organs receiving exosomes as indicated**. U6 served as an internal control and data are expressed as mean ± SEM. ns, not significant, * p<0.05, ** p<0.01, between groups. Error bars represent the SEM for n=3.


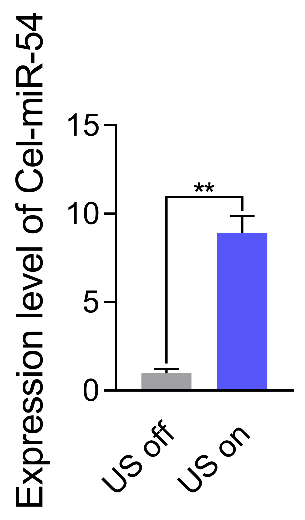


**Figure S3 Expression level of cel-miR-54 in OAT with or without ultrasound irradiation.** U6 served as an internal control and data are expressed as mean ± SEM. *** p<0.001 between groups. Error bars represent the SEM for n=3. US, ultrasound.


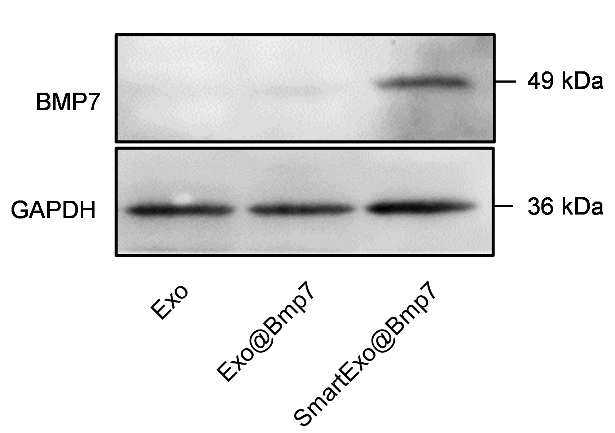


**Figure S4 Western blot analysis of BMP7 in adipose tissue of mice treated with Exo, Exo@Bmp7 and SmartExo@Bmp7.** GAPDH served as an internal control.


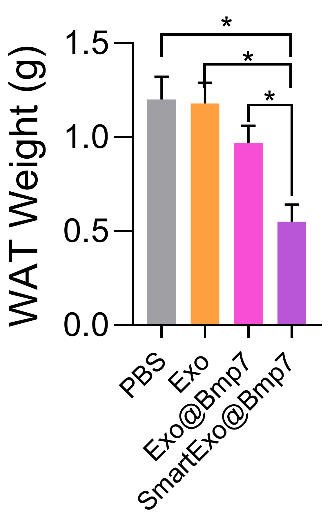


**Figure S5 Average OAT weights of mice treated with PBS, EXO, Exo@BMP7 and SmartExo@BMP7.** All data are expressed as mean ± SEM. * p<0.05 between groups. Error bars represent the SEM for n=3.

**Supplemental Tables**

**Table S1. Ultrasound irradiation parameters.**

| Type | Frequency (MHz) | Intensity (W/cm^2^) | Irradiation time (s) | Duty Cycle |
| --- | --- | --- | --- | --- |
| RAW264.7 | 1 | 0.1 | 30 | 20% |
| Mouse | 1 | 2 | 180 | Continue |

**Table S2. Primers used in the study.**

| miRNA mimics | | |
| --- | --- | --- |
| *cel-miR-54* | Sense | AGGAUAUGAGACGACGAGAACA |
|  | Antisense | UUCUCGUCGUCUCAUAUCCUUU |
| qPCR Primers | | |
| *cel-miR-54* | Forward | 5’-AGGATATGAGACGACGAGAACA-3’ |
|  | Reverse | Provided in the kit |
| *U6b* | Forward | 5’-CTCGCTTCGGCAGCACA-3’ |
|  | Reverse | Provided in the kit |
| *Bmp7* | Forward | 5’-GGAGCGATTTGACAACGAGACC-3’ |
|  | Reverse | 5’-AGTGGTTGCTGGTGGCTGTGAT-3’ |
| *Ucp1* | Forward | 5’-AGGCTTCCAGTACCATTAGGT-3’ |
|  | Reverse | 5’-CTGAGTGAGGCAAAGCTGATTT-3’ |
| *Gapdh (mouse)* | Forward | 5’-AGGTCGGTGTGAACGGATTTG-3’ |
|  | Reverse | 5’-TGTAGACCATGTAGTTGAGGTCA-3’ |
| *GAPDH (human)* | Forward | 5’-CAATGACCCCTTCATTGACC-3’ |
|  | Reverse | 5’-GACAAGCTTCCCGTTCTCAG-3’ |
